# Totally thoracoscopic surgery for the treatment of atrial septal defect without of the robotic Da Vinci surgical system

**DOI:** 10.1186/1749-8090-8-119

**Published:** 2013-05-01

**Authors:** Gaoli Liu, Yanli Qiao, Liming Ma, Liangchun Ni, Shanguang Zeng, Qingchen Li

**Affiliations:** 1 Department of Cardiovascular Surgery, The Affiliated Hospital of Jining Medica University, Shandong Provincial, 79#, Guhuai Road, Jining, 272029, P. R. China; 2 Shandong Provincial Key Laboratory of Cardiac Disease Diagnosis and Treatment, 79#, Guhuai Road, Jining, 272029, P. R. China

**Keywords:** Thoracoscopy, Minimally invasive cardiac surgery, Atrial septal defect, Congenital heart disease

## Abstract

**Background:**

More and more surgeons and patients focus on the minimally invasive surgical techniques in the 21st century. Totally thoracoscopic operation provides another minimal invasive surgical option for patients with ASD (atrial septal defect). In this study, we reported our experience of 61 patients with atrial septal defect who underwent totally thoracoscopic operation and discussed the feasibility and safety of the new technique.

**Methods:**

From January 2010 to October 2012, 61 patients with atrial septal defect underwent totally thoracoscopic closure but not traditional median sternotomy surgery. We divided the 61 patients into two groups based on the operation sequence. The data of group A (the first 30 cases) and group B (the last 31 cases). The mean age of the patients was 35.1 ± 12.8 years (range, 6.3 to 63.5 years), and mean weight was 52.7 ± 11.9 kg (range, 30.5 to 80 kg). Mean size of the atrial septal defect was 16.8 ± 11.3 mm (range, 13 to 39 mm) based on the description of the echocardiography.

**Results:**

All patients underwent totally thoracoscopy successfully, 36 patients with pericardium patch and 25 patients were sutured directly. 7 patients underwent concomitant tricuspid valvuloplasty with Key technique. No death, reoperation or complete atrioventricular block occurred. The mean time of cardiopulmonary bypass was 68.5 ± 19.1 min (range, 31.0 to 153.0 min), the mean time of aortic cross-clamp was 27.2 ± 11.3 min (range, 0.0 to 80.0 min) and the mean time of operation was 149.8 ± 35.7 min (range, 63.0 to 300.0 min). Postoperative mechanical ventilation averaged 4.9 ± 2.5 hours (range, 3.5 to 12.6 hours), and the duration of intensive care unit stay 20.0 ± 4.8 hours (range, 15.5 to 25 hours). The mean volume of blood drainage was 158 ± 38 ml (range, 51 to 800 ml). No death, residual shunt, lung atelectasis or moderate tricuspid regurgitation was found at 3-month follow-up.

**Conclusion:**

The totally thoracoscopic operation is feasible and safe for patients with ASD, even with or without tricuspid regurgitation. This technique provides another minimal invasive surgical option for patients with atrial septal defect.

## Background

Video-assisted thoracoscopic surgical techniques are used safely and widely in treatment of patients with congenital heart diseases now [1,2]. It is a great technological advance from video-assisted thoracoscopic surgery for patent with ductucs atreriosus interruption and vascular ring division to totally thoracoscopic surgery for ASD with or without the aid of robotic Da Vinci surgical system [3-6]. However, the Da Vinci surgical system is not used widely in cardiac surgery area. Totally thoracoscopic surgery without the assisted of the robotically surgical system, which can be performed easily, provides an alteration for patients with ASD and other congenital heart diseases.

Since January 2010, 61 consecutive patients underwent ASD closing with the totally thoracoscopic technique in our institution. In this study, we reported the mid-term follow-up results in these patients and our experience of this technique.

## Materials and methods

The Clinical Review Board of the Affiliated Hospital of Jining Medical University approved this study and written informed consent and operation related permission, including the use of their pictures were obtained from each patient. We excluded patients who required ventricular septal defect closing or other concomitant cardiac procedures, but including the patients who underwent concomitant tricuspid valvuloplasty. For this operation we selected the patients must met: (1) more than 6 years old and the weight 30 kg or more. (2) the pulmonary systolic pressure no more than 50 mmHg estimated by the echocardiography. (3) without left superior vena cava. (4) no previous operaion of the right chest or chronic lung disease. (5) without femoral vein and artery malformation or thrombosis.

From January 2010 to October 2012, 61 patients with atrial septal defect underwent totally thoracoscopic closure but not traditional median sternotomy surgery. The mean age of the patients was 35.1 ± 12.8 years (range, 6.3 to 63.5 years), and mean weight was 52.7 ± 11.9 kg (range, 30.5 to 80 kg). Mean size of the atrial septal defect was 16.8 ± 11.3 mm (range, 13 to 39 mm) based on the description of the echocardiography.

All of the patients were given general anesthesia and double-lumen endotracheal intubation. The respiration rate was set between 18 and 25 breaths/min, and tidal volume was between 8 and 10 ml/kg. Routine monitoring included blood pressure, pulse oxygen saturation and electrocardiogram reading. All the patients were supine position with the right side of the body elevated to 15° to 20° to facilitated the operation.

**Figure 1:**
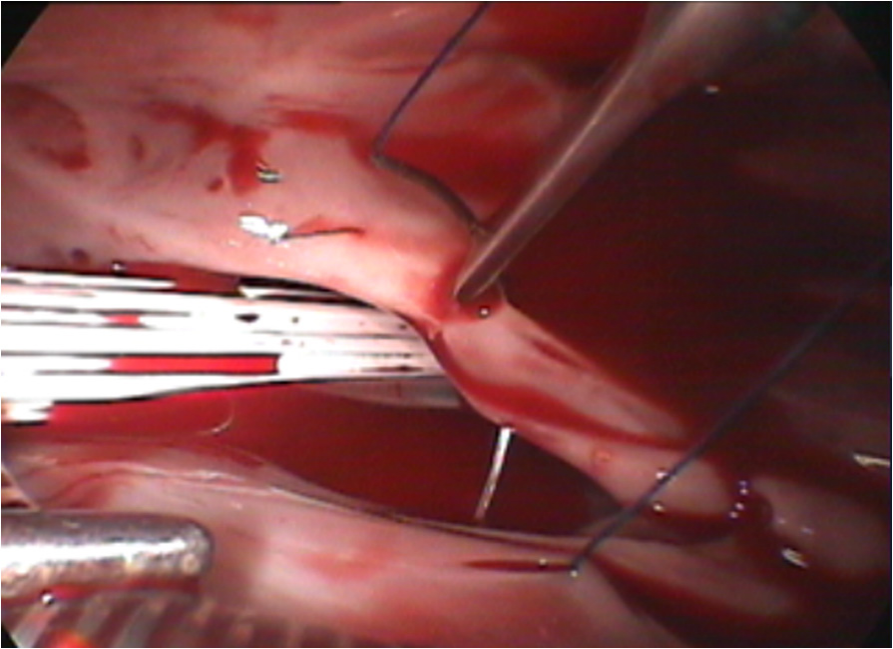
ASD closing.

**Figure 2:**
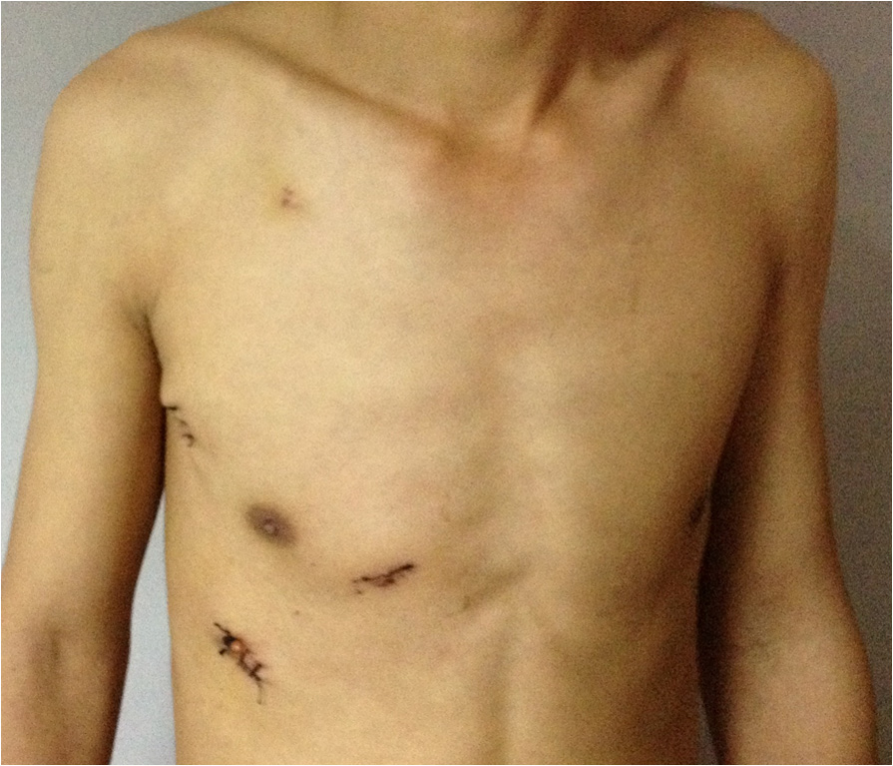
The incisions in the chest.

Three incisions approximately 1.5 cm long were made on the right anterior axillary line at the sixth intercostal space (incision 1), the right midaxillary line at the third intercostal space (incision 2) and the parasternal line at the fourth intercostal space (incision 3), respectively (Figure 1). A plastic retractor was placed through the incisions to make the tissue open and facilitate the procedure. Incision 1 was for the placement of the thoracoscopy (Shenda, China). Incision 2 was for the insertion of the aortic cross-clamping forceps and tissue forceps, using the left hand. Incision 3 was for the placement of the surgical instruments, such as scissor and needle holder, using the right hand. After the systemic heparinization, the femoral vein and artery were accessed through an oblique incision which 1 cm above the inguinal ligament. Femoral artery cannulation was performed with a 21 F cannula (ZO, Kangxin, China) and femoral venous cannulation with a 24/29 F double-lumen catheter (Kewei, China). The single two-stage femoral venous cannula drains blood simultaneously both the superior and the inferior vena cava during the operation [7]. The pericardium was incised, and the free edges suspended conventional under the guidance of the thoracoscopy. The cardiopulmonary bypass was established with standard procedure as reported [8,9]. A long aotic aross-clamping forceps was placed directly on the ascending aorta, and a cannula for delivery of cardioplegia was inserted to the aortic root both through the incision 2. After the heart was arrested, the right atrium was opened along the site parallel to the atrioventricular groove by the forceps and scissor through the incision 2 and incision 3, respectively. The ASD was closed with 4–0 prolene (Ethicon) directly or with self-pericardium patch based on the size of the defect (Figure 2). Deairing was done from the cannula for delivery of cardioplegia and the ASD defect before knotting by lung inflation. The right atrium was sutured by 4–0 prolene (Ethicon) after the aortic clamp released and body warmed. After withering from cardiopulmonary bypass, the cannulations were pulled out. A chest tube was placed in the right plural space from incision 1 for drainage effusion and air.

### Statistical analysis

Spss version 13.0 software was used for statistical analysis. Quantitative variables were expressed as means ± standard deviations.

## Results

All patients underwent totally thoracoscopy successfully, 36 patients with pericardium patch and 25 patients were sutured directly. 7 patients underwent concomitant tricuspid valvuloplasty with Key technique. No death, reoperation or complete atrioventricular block occurred. The mean time of cardiopulmonary bypass was 68.5 ± 19.1 min (range, 31.0 to 153.0 min), the mean time of aortic cross-clamp was 27.2 ± 11.3 min (range, 0.0 to 80.0 min) and the mean time of operation was 149.8 ± 35.7 min (range, 63.0 to 300.0 min). Postoperative mechanical ventilation averaged 4.9 ± 2.5 hours (range, 3.5 to 12.6 hours), and the duration of intensive care unit stay 20.0 ± 4.8 hours (range, 15.5 to 25 hours). The mean volume of blood drainage was 158 ± 38 ml (range, 51 to 800 ml). No death, residual shunt, lung atelectasis or moderate tricuspid regurgitation was found at 3-month follow-up .

We have divided the 61 patients into two groups based on the operation sequence. The data of group A (the first 30 cases) and group B (the last 31 cases) is presented in the Table 1. The aortic crossclamp, CPB (cardiopulmonary bypass) and operation time of group A are much less than the time of group B. The incidence of lung atelectesis is also better in group A compared to group B. No complications such as wound infection, pneumothorax, poor femoral wound healing, femoral vein or artery bleeding. 1 patient occurred chest incision poor healing, and cured through strengthening dressing change and debridement suture. 3 patients got left lobe atelectasis, and cured by directly suction from trachea and lung exercises. All patients had a re-evaluation by echocardiography, chest X-ray and electrocardiogram after 3 month of the operation. No death, residual shunt and lung atelectasis or moderate tricuspid regurgitation was observed.

**Table 1:** The operation results of the two groups

	**Group A**	**Group B**	
Aotic crossclamp time	36	19	P < 0.05
CPB time	73	56	P < 0.05
Operation time	182	139	P < 0.05
Lung atelectesis	3	0	P < 0.05

## Discussion

Video-assisted thoracoscopic surgical techniques are used widely in cardiac surgery now. It is a great technological advance from video-assisted thoracoscopic surgery for patent ductucs atreriosus interruption and vascular ring division to totally thoracoscopic surgery for ASD with or without the aid of robotic Da Vinci surgical system. Totally thoracoscopic surgery with the assistance of robotic Da Vinci surgical system is feasible [10], but it is a big problem for lots of hospitals because of the expensive cost for the robot and its training [11]. Totally thoracoscopic techniques without the aid of the Da Vinci system in cardiac operation now become a reality with the technological development. Totally thoracoscopic techniques for cardiac surgery only need three small incisions with 1.0-2.5 cm each in length [12]. The techniques avoid the conventional midline sternotomy and change the opinion that a direct vision should be needed in cardiac surgery and also provide a cosmetic cut for patients.

We performed totally thoracoscopic surgery for ASD since 2010 with lots of experience of zoopery, and produced excellent results. The mean time of aortic cross-clamp was 27.2 ± 11.3 min (range, 0.0 to 80.0 min) and the mean time of operation was 149.8 ± 35.7 min (range, 65.0 to 300.0 min). Reports on robotic assisted thoracoscopic surgery on ASDs closing showed mean aortic cross-clamp times of 32 to 93 minutes [13-15]. Our operation results are satisfied compared with those of the reported groups. The aortic crossclamp, CPB and operation time of group A are much less than the time of group B. The incidence of lung atelectesis is also better in group A compared to group B. We consider that the operators could master this technique easily and quickly. All patients had a re-evaluation by echocardiography, chest X-ray and electrocardiogram after 3 month of the operation. No residual shunt and lung atelectasis were found. We selected the patients more than 6 years old and the weight 30 kg or more considering the diameter of femoral vessels and the early period of this technique applied in our hospital. The reason of poor chest incision healing in 1 patient is in relation to the long time tight press of the plastic retractor to the incision during the operation, and exactly why 3 patients occurred left lobe atelectasis in group A is not sure, but it might be in relation to the heat press to the left lung because of the right side elevated supine position during the operation and inadequate lung inflation after the operation. Administering the measures of direct suction from trachea is an effective way for atelectasis, it can remove secretion that obstruct the air way efficiently, keep respiratory tract unobstructed and make atelectasis inflated. Chest physiotherapy and positive end-expiratory pressure both are the useful adjunct to treatment for the atelectasis [13]. The lung atelectasis did not occur in group B and this maybe in relation to the technological improvement of our intensive care.

The merits of the totally thoracoscopic surgery are mainly in less pain and trauma avoiding the midline sternotomy and the use of the steel wire, less bleeding after the operation with the least drainage volume was only 51 ml and with a more beautiful incision. The conventional operation is a three-dimensional vision that appears to have height, width and depth. The thoracoscopic vision is only a two-dimensional image which lacks the feeling of depth and all the operations are indirectly accomplished with instruments, some aspects should be paid more attention especially in the beginning application of this new technique. Even an intracorporeal knot is one of the technical challenges of thoracoscopic surgical approach [14]. The manipulation of the superior vena cava and inferior vena cava must softly and skillfully, and we performed double purse string suture in the aorta for inserting the cardioplegia canula in every patient. The vascular bleeding no matter the vein or the artery is annoying and increases the risk of the operation. To avoid air embolism and cardiac dysfunction after operation, more attention should be paid to deair and ventricular distention during the operation. The totally thoracoscopic surgery acquire the operator having both the foundation of the conventional cardiac surgery and special training of endoscopy for a good command of two-dimensional image. It is necessary for a period of zoopery before the application of this new technique.

## Conclusions

In conclusion, we reported 61 consecutive patients of totally thoracoscopic surgical treatment for ASD without the aid of robotic system. The results were encouraging and need to be confirmed by larger series. We did not perform this technique in younger children because of the early time in our hospital. We believe that it will be applied in younger children and other cardiac diseases in future.

## Abbreviations

ASD: Atrial septal defect; CPB: Cardiopulmonary bypass.

## Competing interests

The authors declare that they have no competing interests.

## Authors’ contributions

GL carried out the clinical studies and drafted the manuscript. YQ carried out the operations. LM participated in the design of the study. LN participated in the design of the study and performed the statistical analysis. SZ conceived of the study, and participated in its design. QL helped to draft the manuscript. All authors read and approved the final manuscript.
